# Adsorption of Polylactic-co-Glycolic Acid on Zinc Oxide Systems: A Computational Approach to Describe Surface Phenomena

**DOI:** 10.3390/nano14080687

**Published:** 2024-04-16

**Authors:** Elaheh Mohebbi, Eleonora Pavoni, Cristina Minnelli, Roberta Galeazzi, Giovanna Mobbili, Simona Sabbatini, Pierluigi Stipa, Mir Masoud Seyyed Fakhrabadi, Emiliano Laudadio

**Affiliations:** 1 Department of Science and Engineering of Matter, Environment and Urban Planning, Marche Polytechnic University, 60131 Ancona, Italy; e.mohebbi@staff.univpm.it (E.M.); e.pavoni@staff.univpm.it (E.P.); s.sabbatini@staff.univpm.it (S.S.); p.stipa@staff.univpm.it (P.S.); 2 Department of Life and Environmental Sciences, Marche Polytechnic University, 60131 Ancona, Italy; c.minnelli@staff.univpm.it (C.M.); r.galeazzi@staff.univpm.it (R.G.); g.mobbili@staff.univpm.it (G.M.); 3 School of Mechanical Engineering, College of Engineering, University of Tehran, Tehran P.O. Box 14155-6619, Iran; mfakhrabadi@ut.ac.ir

**Keywords:** DFT, PLGA, ZnO, electrical properties, adsorption, Young’s modulus, bulk modulus

## Abstract

Zinc oxide and polylactic-co-glycolic acid (ZnO-PLGA) nanocomposites are known to exhibit different biomedical applications and antibacterial activity, which could be beneficial for adding to wound dressings after different surgeries. However, possible cytotoxic effects along with various unexpected activities could reduce the use of these prominent systems. This is correlated to the property of ZnO, which exhibits different polymeric forms, in particular, wurtzite, zinc-blende, and rocksalt. In this study, we propose a computational approach based on the density functional theory to investigate the properties of ZnO-PLGA systems in detail. First, three different stable polymorphs of ZnO were considered. Subsequently, the abilities of each system to absorb the PLGA copolymer were thoroughly investigated, taking into account the modulation of electrical, optical, and mechanical properties. Significant differences between ZnO and PLGA systems have been found; in this study, we remark on the potential use of these models and the necessity to describe crucial surface aspects that might be challenging to observe with experimental approaches but which can modulate the performance of nanocomposites.

## 1. Introduction

Over the last decade, a significant effort has been dedicated to developing micro- and nanoparticles (NPs) for drug delivery since they allow the distribution of therapeutically active drug molecules only to the target site of action without affecting healthy organs and tissues and avoid toxic processes [1,2]. Many different polymers have been employed for drug delivery in producing biocompatible and biodegradable NPs; in particular, the polyester, poly (DL-lactide-co-glycolide) (PLGA) is a copolymer approved by the Food and Drug Administration (FDA) for medical and pharmaceutical applications, and for this reason, it is one of the most famous polymers adopted in biomedical fields [3]. Its biodegradation rate can be modulated by changing the percentage of lactic acid since the degradation process is prolonged when more lactic acid is present. PLGA, with a 50:50 composition, is formed by equal proportions of lactic and glycolic acids and represents the most popular composition in nanomedicine due to its rapid biodegradation rate of approximately 50 days [4].

**Figure 1:**
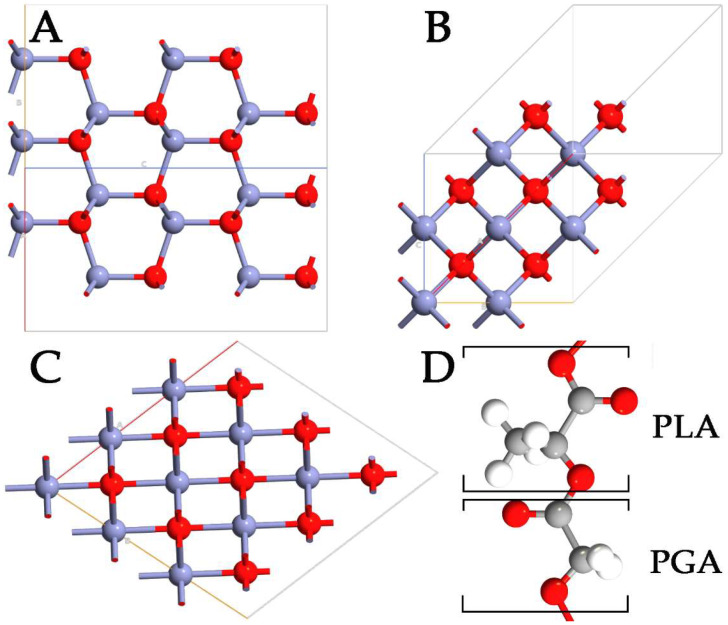
Representation of WZ- (**A**), ZB- (**B**), RS- (**C**) ZnO polymorphs, and PLGA (**D**) polymers. The monomers of lactic and glycolic acid are marked with PLA and PGA, respectively. Zn, O, C, and H are highlighted in purple, red, gray, and white, respectively.

Despite the advantages of using polymers in drug delivery, it is difficult to control the size of NPs, compromising the capabilities and the behavior of the systems. To overcome this problem, the physicochemical properties of polymer NPs can be modulated, including small size and very large specific surface area. These modifications can significantly enhance the bioavailability of the systems while, at the same time, minimizing drug toxicity. In this context, inorganic antibacterial NPs offer some benefits in overcoming major problems related to the treatment process, such as reducing the dosage of antibacterial agents, minimizing side effects, overcoming bacterial resistance, and lowering the overall cost of the fabrication process [5,6]. Among the wide range of inorganic systems used, zinc oxide (ZnO) stands out as a multifunctional material. In its ground state, ZnO can be observed in a stable hexagonal wurtzite (WZ) structure (Figure 1A). However, the metastable zinc-blende (ZB) (Figure 1B) and rocksalt (RS) (Figure 1C) polymorphs of ZnO can also be detected [7,8]. ZnO is one of the most studied semiconductors from the group II-VI family [9,10], with a large and direct bandgap energy of 3.37 eV. Besides its extraordinary optical, catalytic, semiconducting, and piezoelectric properties, ZnO is a promising material for a variety of biological applications because of its chemical stability, antimicrobial activity, high biocompatibility, and nontoxicity to human cells [11]. Moreover, zinc is an essential mineral for human health and is used in the form of ZnO in the daily supplement for zinc. Thanks to the properties reported, ZnO NPs can be used for cellular imaging [12], biodetection [13], drug delivery [14], and various kinds of therapy [15]. ZnO has a wide range of different nanostructures already used in numerous commercial products, such as sunscreen products, different types of sensors, industrial coatings, and antibacterial agents [16]. Specifically, ZnO NPs exhibit a broad spectrum of antibacterial activities towards Gram-positive and Gram-negative bacteria, including major food-borne pathogens, which are mainly attributed to the generation of reactive oxygen species (ROS) on the surface.

In recent years, much attention has been paid to the preparation of organic/nanosized inorganic particles for different types of applications, especially in medicine and pharmacy [17]. The properties of nanocomposites are greatly influenced by both the dispersing degree of NPs in the base polymers and the interfacial adhesion between the inorganic and organic components [18]. ZnO has been already incorporated in a variety of different polymers such as poly (methyl methacrylate) [19], polystyrene [20], polyacrylonitrile [21], and polyurethane [22], and the results have shown that the prepared polymer/ZnO composites presented better performances compared to the polymers alone. This means that the importance of studying the ZnO–polymer interactions is of primary importance for many applications. Gedda et al. [19] introduced nanoparticle-assisted liquid–liquid microextraction based on ZnO nanoparticles modified with polymethyl methacrylate to extract pathogenic bacteria. Amna et al. [22] fabricated the ZnO–polyurethane nanocomposite by an electrospinning technique and evaluated in vitro the viability, attachment, and proliferation of NIH 3T3 mouse fibroblast cells on the ZnO–polyurethane; their cytotoxicity experiments indicated that hybrid nanofibers represent a promising biomaterial for tissue engineering applications. Chen et al. [21] presented self-standing polyacrylonitrile–ZnO/Ag composite, considering different ZnO morphologies that can be applied in smart textiles as water purification filters or antibacterial wear.

Although nanocomposites can be prepared by simply mixing NPs with base polymers, the dispersing degree of NPs and the interfacial linkage were insufficient to obtain desirable material properties [23]. Moreover, the use of different ZnO polymorphs can lead to different complexes, which could exhibit unexpected instability or mechanical and electronic inefficiency. For this reason, with the aim of shedding light on short-range phenomena based on the generation of PLGA-ZnO NPs to provide efficient systems for drug delivery applications, a detailed computational approach based on the density functional theory (DFT) was used in this work. In detail, the differences between WZ, ZB, and RS ZnO polymorphs have been elucidated, calculating the lattice parameters and the band structures for each system. Then, the efficiency of each polymorph to absorb PLGA (Figure 1D) oligomers has been investigated considering the energy formation and the variations in density of the states (DOS). In addition, the modulation of the optical properties has been considered in terms of the simulated absorption spectra for each system with and without PLGA. Finally, the mechanical properties of each ZnO-PLGA system have been calculated by means of Young’s and bulk moduli. The final purpose of this work is to investigate all the phenomena difficult to observe from an experimental point of view, providing essential information useful as guidelines to fabricate more efficient and healthier ZnO-PLGA NPs for drug delivery applications.

## 2. Materials and Methods

The three ZnO polymorphs were optimized with a DFT approach, and then, the partial density of the states (PDOS) and the adsorption of PLGA on the various surfaces were calculated. Quantum Atomistic Toolkit (Q-ATK) [24] software was used to optimize the hexagonal *P6₃mc* space group (WZ), cubic $F\overset{-}{4}3m$ space group (ZB), and cubic $Fm\overset{-}{3}m$ space group (RS) of ZnO. The optimized structures of the polymorphs were then used to generate polymer–ZnO systems and to simulate the adsorption of PLGA and its mechanical properties. The single particle wave functions were expanded using the plane wave (PW) method in the PW basis set [25] for all the Zn, O, C, and H entities. Perdew–Burke–Ernzerhof’s (PBE) GGA density functional theory [26] was used for the electron exchange–correlation (xc) of energy. For each element, the ionic cores were described with norm-conserving (NC) PseudoDojo (PDj) pseudopotentials [27]. Regarding the valence electrons, the 3d^10^ and 4s^2^ electrons of Zn and the 2s^2^ and 2p^4^ electrons of O were taken as the valence electrons, as well as for the 2s^2^ and 2p^2^ electrons of C, and the only 1S^1^ electron of H. All ZnO systems were modeled using periodic boundary conditions (PBCs) along all axes, avoiding problems with the boundary effects caused by the finite size and, at the same time, maintaining high accuracy, guarantying the feasibility of the simulations, and reducing the number of explicit atoms in the simulation box. To calculate the interface phenomena, PBC conditions were used along the x and y in-plane axes, interrupting periodicity along the out-of-plane *z*-axis. The energy cut-off was fixed at 1200 eV, and the Brillouin-zone integration was performed over a 15 × 15 × 15 k-points grid for the modeled polymorphs [28]. These parameters assured the total energy convergence of 5.0 × 10^−6^ eV/atom, the maximum stress of 2.0 × 10^−2^ GPa, and the maximum displacement of 5.0 × 10^−4^ Å. The polarization in the polymorphs was calculated using the modern theory of polarization [29] and the Berry phase operator. In detail, it was obtained by the sum of the electronic (*Pe*) and ionic (*Pi*) contributions.

The electronic one (*Pe*) was calculated as in Equation (1):

(1) $$P_{e}=-\frac{2\left|e\right|i}{{\left(2\pi \right)}^{3}}\int_{A}dk_{⊥}\sum_{n=1}^{M}\int_{0^{\left\langle U_{k,n}\left|\frac{\partial }{\partial k}\right|u,<,n\right\rangle }}^{G}dk$$

where the sum runs over occupied bands and *k*, and the direction of polarization is parallel to each other. The *G* term is a reciprocal lattice vector in the same direction. The states *U_k,n_* are the cell-periodic parts of the Bloch functions *ψ _k,n_ (r) = u _k,n_ (r) e ^ikr^*. The last integral is known as the Berry phase [30]. The ionic contribution (Pi) was calculated using a simple classical electrostatic sum of point charges, as reported in Equation (2):

(2) $$P_{i}=\frac{\left|e\right|}{\Omega }\sum_{\nu }Z_{ion}^{v}r^{v}$$

where Ω is the unit cell volume, *Z^v^_ion_* is the valence charge, and *r^ν^* is the position vector of the *ν* atom.

The optical absorption coefficient is related to the extinction coefficient through Equation (3):

(3) $$\alpha_{a}=2\frac{\omega }{C}Κ$$

Κ is the extinction coefficient, and it was derived by the real (ε_r_ (ω)) and the imaginary (ε_i_ (ω)) part of the dielectric constant following Equations (4) and (5):

(4) $$\varepsilon_{r}\left(\omega \right)=n^{2}-k^{2}$$

(5) $$\varepsilon_{i}\left(\omega \right)=2nk$$

Finally, ε_r_ (ω) was obtained with the following Equation (6):

(6) $$\varepsilon_{r}\left(\omega \right)=1+\frac{1}{\pi }P\int_{0}^{\infty }d\bar{\omega }\frac{\bar{\omega }\varepsilon_{2}\left(\bar{\omega }\right)}{{\bar{\omega }}^{2}-\omega^{2}}$$

And ε_i_ (ω) was found following Equation (7):

(7) $$\varepsilon_{i}\left(\omega \right)=\frac{4\pi^{2}}{\Omega \omega^{2}}\sum_{i\in HOMO,j\in LUMO}\sum_{k}W_{k}{\left|\rho_{ij}\right|}^{2}\delta \left(\varepsilon_{kj}-\varepsilon_{ki}-\hbar \omega \right)$$

where HOMO, LUMO, ω, Ω, Wk, *ρ_ij_* are the valence band, conduction band, photon frequency, volume of the lattice, weight of the *k*-point, and elements of the dipole transition matrix, respectively.

The surface area of each system was created by multiplying the unit cell dimensions of each polymorph 8 times along the x, y, and z axes. Then, 4 repetitions of each ZnO system along z were deleted to ensure there was a reasonable space to model the polymer chains and, at the same time, to maintain the proportions between the axes of the simulation box. The x-y dimensions were 3.72, 3.42, and 3.64 nm, while the thicknesses were 1.88, 1.96, and 1.84 nm for the slabs WZ-, ZB-, and RS-ZnO, respectively. Finally, PLGA chains were built atom by atom in the empty space along the z axis. The polymer chain was relaxed in space, exhibiting periodicity along the x and y axes.

Young’s in-plane moduli of ZnO and ZnO-PLGA systems were computed by the relation of *Y_s_* = *Et*. In this equation, *t* is the thickness, and *E* represents Young’s modulus. The reason that in-plane Young’s modulus was used instead of Young’s modulus was to avoid any conflicts caused by the different thickness values of different polymorphs in the presence of PLGA, which can lead to different values for Young’s moduli of the same structure. The final form of *Y_s_* that was used in this work is expressed as follows (7) [31]:

(8) $$Y_{s}=\frac{1}{A_{0}}\frac{\partial^{2}E_{s}}{\partial \varepsilon^{2}}$$

where *A*_0_ is the cross-sectional area of the unit cell, while *E_s_* is the strain energy, and its second derivative is calculated with respect to the strain *ε*.

Finally, the bulk modulus of the ZnO and ZnO-PLGA structure was calculated by (9) [32]:

(9) $$B=A\left(\frac{\partial^{2}Es}{\partial A^{2}}\right)$$

Finally, the possibility of having some hydroxylated O atoms on the ZnO surfaces was not investigated. The reason for this was that if the simulated environment is not reducing or physiological, then the solvent and ions are not taken into account. Therefore, even if some groups can certainly undergo hydroxylation, such variables are not included in order to better investigate the intrinsic capabilities of the different ZnO polymorphs to adsorb PLGA.

## 3. Results and Discussions

### 3.1. Ground and Excited State ZnO Properties

With the aim of demonstrating the reliability of the computational method, ZnO polymorphs were deeply analyzed in terms of lattice parameters (Table 1), and the results were compared to experimental data reported in the literature. The calculated data validated the chosen approach since the calculated unit cells were in good agreement with the data reported. The WZ ZnO polymorph is composed of two interpenetrating hexagonal close-packed sublattices of Zn^2+^ and O^2^⁻ ions displaced by the length of the cation–anion bond along the c-direction. The lattice constant of this ZnO hexagonal unit was found to be a = 3.25 Å and c = 5.21 Å, with bond lengths of 2.04 Å and bond angles of 105.75°. The ZB polymorph exhibited each Zn^2^⁺ bonded to four equivalent O^2^⁻ atoms to form corner-sharing ZnO₄ tetrahedra. All Zn-O bond lengths were 1.93 Å. The anion O^2^⁻ was bonded to four equivalent Zn^2^⁺ ions to form corner-sharing OZn₄ tetrahedra, with bond angles of 106.02°, and the lattice parameters were found to be a = b = 3.24 Å and c = 5.22 Å. In the RS polymorph, Zn^2^⁺ was bonded to six equivalent O^2^⁻ atoms to form a mixture of edge and corner-sharing ZnO₆ octahedra, which were not tilted. All Zn-O bond lengths were 1.79 Å, the lattice parameters were a = b = c = 4.34 Å, and the bond angles were 78.22°.

**Figure 2:**
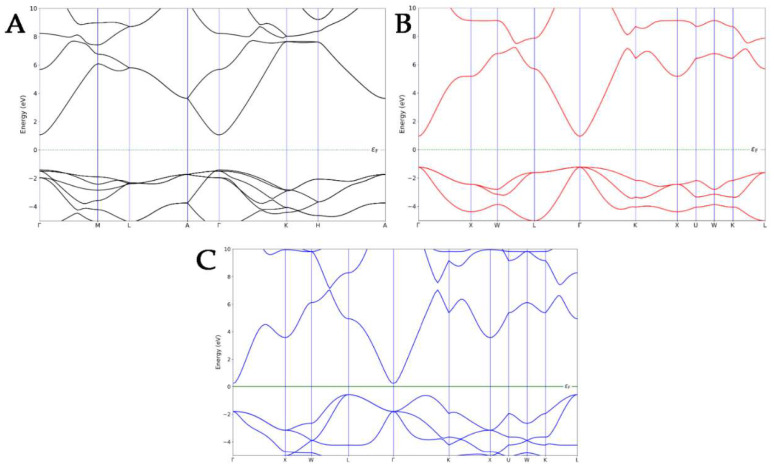
Band structures of WZ- (**A**), ZB- (**B**), and RS- (**C**) ZnO polymorphs.

Before focusing on the electronic properties of the nanocomposites, the band structures of each ZnO pure polymorph were calculated and then compared to the experimental results in the literature (Figure 2). The electronic band structure of WZ exhibited a direct bandgap with a conduction band minimum (CBM) and the valence band maximum (VBM) at the same Γ-point, as confirmed by the experimental data [36,37]. A direct bandgap was also observed in the Γ-point for the ZB polymorph, which is in line with the literature [38]. Finally, in the case of an RS-type structure, an indirect bandgap was observed, with the VBM that shifted at the L-point and CBM, which still occurred at the Γ-point [39]. The ZB ZnO exhibited the smallest calculated bandgap, while the highest was observed for the WZ polymorph, underlining the same trend observed in the literature [40].

### 3.2. Structural and Electrical Variation Induced by PLGA Adsorption

**Figure 3:**
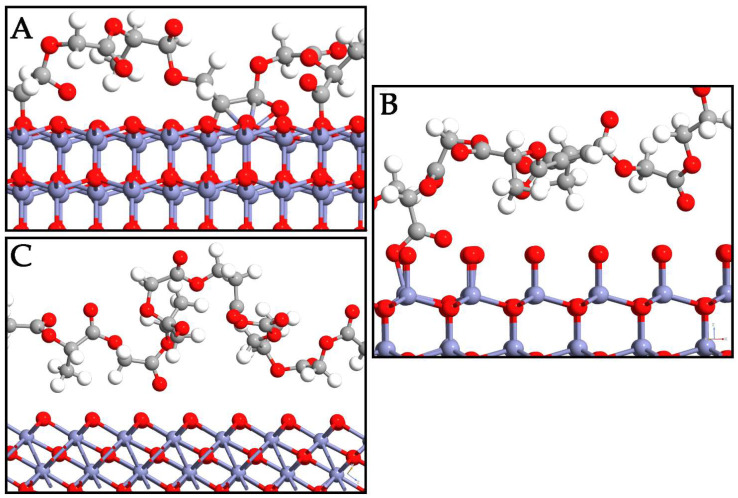
Depiction of PLGA adsorption on WZ- (**A**), ZB- (**B**), and RS- (**C**) ZnO surfaces. Zn, O, C, and H are reported in purple, red, gray, and white, respectively.

In this section, the interaction mode of PLGA on the ZnO surfaces is deeply investigated. Different displacements of copolymer are taken into account to determine the most probable adsorption sites on the surfaces. To avoid losing probable three-dimensional accommodations, the PLGA was manually displaced in different starting positions on each polymorph surface. As a result of the optimization, the most probable PLGA arrangements after adsorption directly involved its methyl and hydroxyl groups on the three interfaces (Figure 3). The polymer was oriented in a horizontal direction to the WZ ZnO surface, while important variations in the PLGA location were detected in the ZB and RS polymorphs. Indeed, the oxygen on the *P6₃mc* surface exhibited higher reactivity with respect to the other polymorphs, and this phenomenon was due to the tensile stress of this phase, which could be attributed to the forced geometrical configuration of the bridging oxygen when PLGA was present. This led to a decrease in the Zn–O bond lengths from 1.87 Å to 1.81 Å and an increase in the Zn–O–Zn angle from ~105° to ~110°, moving from the WZ- to ZB-PLGA system.

The calculated binding affinity (Table 2) between PLGA and WZ ZnO was −1.92 eV, confirming the PLGA chemisorption. The O atoms of the WZ surface were directly involved in the interaction with the PLGA, while the C atoms of the polymer and the Zn ions exhibited different contacts between them. The bond lengths (Table 2) of the PLGA atoms closest to the WZ ZnO surface were 1.42 Å (O_ZnO_-C_polymer_), 2.03 Å (Zn_ZnO_-C_polymer_), and 1.81 Å (Zn_ZnO_-O_polymer_). In the ZB ZnO-PLGA composite, the binding affinity was −0.61 eV and the bond length was 1.37 Å. This means that closer atoms were present on the interface between the two systems, but the Zb-PLGA interaction resulted in being poor and not enough to guarantee polymer adsorption. The methyl groups of PLA units did not allow the polymer chain to interact with the ZB surface, which exhibits axial O in this configuration, resulting in a low number of ZnO-PLGA contacts. Only a very few O atoms of PLGA were able to interact with ZB ZnO, with a Zn_ZnO_-O_polymer_ distance of 1.87 Å, and this is the reason for the decreased binding affinity.

The RS ZnO-PLGA system showed a similar trend to the ZB system, with a positive binding affinity (0.03 eV) and a lack of stable bonds between the systems. The configuration of the polymer resulted in a more distorted orientation along the *z*-axis with respect to ZnO. From these results, the RS polymorph failed to adsorb the PLGA. On the other hand, the WZ polymorph was the best choice in this regard since the PLGA-WZ interactions were stronger than PLGA-ZB and PLGA-RS despite the increased bond length.

**Figure 4:**
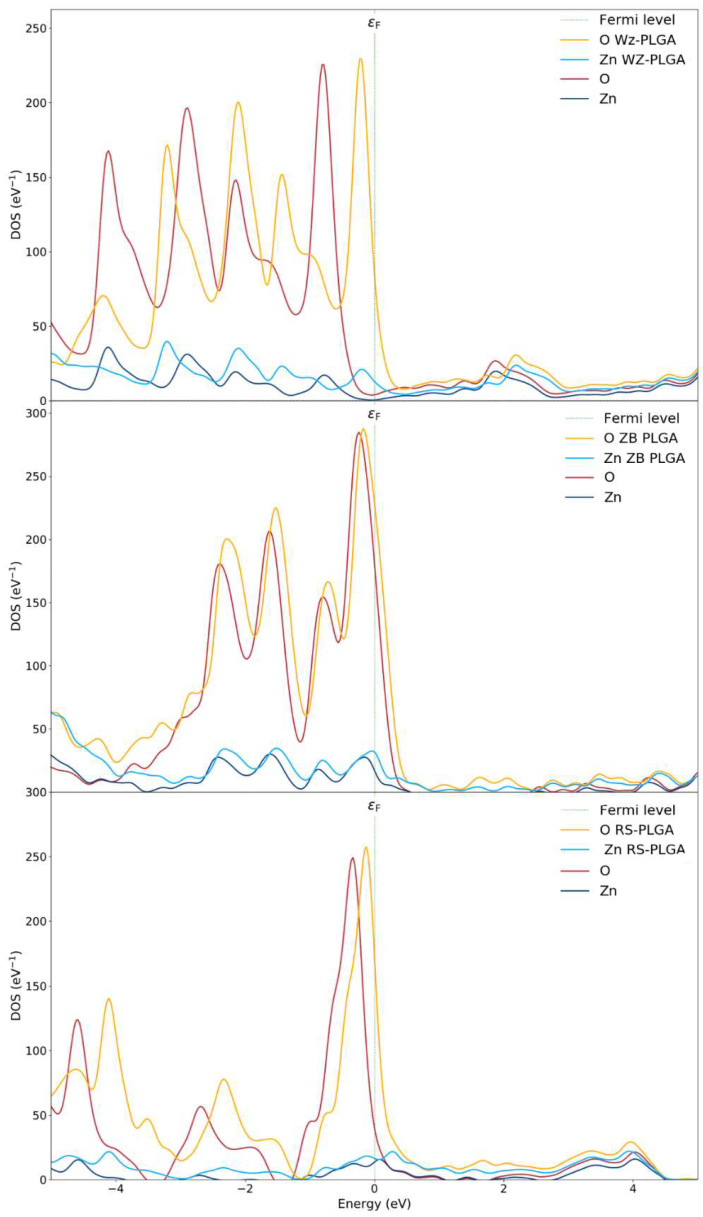
Density of states of WZ and WZ-PLGA, ZB and ZB-PLGA, and RS and RS-PLGA.

The partial density of states (PDOSs) of the ZnO polymorphs and the PLGA-ZnO systems was calculated to describe the effects of PLGA adsorption on the electron transfer phenomena (Figure 4). The PDOS near the Fermi level was due to the interaction of the copolymer with the WZ surface, determining an increase in the VBM up to 1 eV compared to the WZ ZnO without a polymer. This shift is attributed to the increase in the Coulomb potential given by the charge transfer. An evident variation in the electronic structure of ZnO can be associated with the PLGA adsorption on the WZ polymorph since the polymer interacts at different points of the WZ surface. Therefore, the PDOS spectra of the *P6₃mc* polymorph exhibit evident alterations before and after the adsorption processes, and this is certainly correlated to the chemical interaction with PLGA.

From these results, the electronic properties and the adsorption process are linked to the charge transfer, and this is the main reason for the variations in the electronic properties changing the polymorph. As a confirmation, the spectra for both pure ZB- and RS- polymorphs and with PLGA were approximately the same. In fact, the PDOS of the PLGA binding on the ZnO surfaces of $F\overset{-}{4}3m$ and $Fm\overset{-}{3}m$ polymorphs showed no notable shifts apart from an increased magnitude for the oxygen atoms due to the inclusion of both O_PLGA_ and O_ZnO_. This result confirms a weak interaction and no evident hybridizations for PLGA on the ZB system. For this reason, a very small shift in valence bands was observed. Finally, the RS-PLGA exhibited no shifts, and this confirmed the inability of this polymorph to adsorb the copolymer. According to these results, the stable complex of PLGA on the WZ surface, together with the efficient charge transfer, suggest the use of *P6₃mc* as the only ZnO polymorph for efficient polymer adsorption.

### 3.3. Optical Modifications Induced by PLGA Adsorption

**Figure 5:**
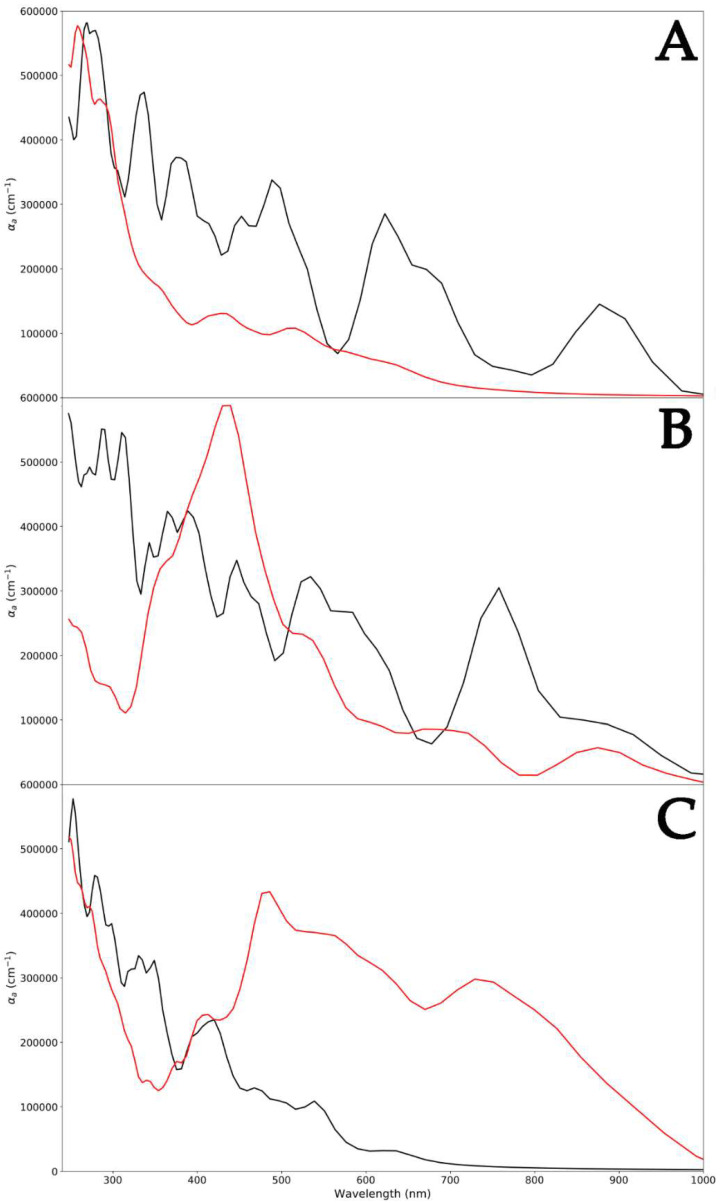
Absorption spectra of WZ (black) and WZ-PLGA (red) (**A**); ZB (black) and ZB-PLGA (red) (**B**); and RS (black) and RS-PLGA (red) (**C**).

Since different ZnO surfaces exhibited peculiar capabilities to absorb PLGA, a polymer-induced change in the optical absorption spectrum was certainly expected (Figure 5). For each ZnO polymorph, high absorption was found at 250 nm. Two absorption peaks were found at 378 nm and 650 nm, and this is characteristic of the WZ polymorph (Figure 5A) [41]. A red shift of the same peak was detected for the ZB ZnO, and this could be attributed to the development of shallow levels in this polymorph (390 nm and 780 nm) (Figure 5B) [42]. This shift in the absorption peaks to higher wavelengths also indicated a decrease in the optical bandgap [43,44]. Indeed, the computed optical bandgap value, as shown in Figure 2, was found to be 3.38 eV for WZ ZnO and 3.29 eV for the ZB polymorph. The decreased optical bang gap could be due to the quantum confinement effects. Finally, the optical bandgap calculated for RS ZnO was 3.44 eV, indicating an opposite trend with respect to that exhibited by ZB. In fact, in this case, a blue shift in the absorption peaks with respect to the WZ polymorph was observed (290 nm and 410 nm) (Figure 5C).

Regarding pure PLGA, the UV–vis absorption spectrum revealed bands at 250 nm [45], as reported in the literature. Focusing on the WZ-PLGA system, the absence of bands attributed to the WZ polymorph in the spectrum indicated the encapsulation of the PLGA shell and on the hexagonal P6₃mc surface. This was in line with experimental evidence in which the occurrence of this characteristic absorption band ascertained the presence of the ZnO-PLGA hybrid polymeric nanocomposite [46]. In the case of ZB-PLGA, the spectrum was characterized by a red shift in adsorption to 430 nm, and after the interaction of PLGA with the $F\overset{-}{4}3m$ surface, absorption bands appeared at 520 and 700 nm. Moreover, a large absorption band ranging between 440 and 480 nm was observed, and this might be ascribed to the varied charge centers displayed by the numerous polymer lateral groups along the ZnO surface. Regarding the RS-PLGA system, another red shift in peaks was detected. The previously reported signals of 430, 520, and 700 nm for ZB-PLGA moved to 490, 580, and 750 nm in $Fm\overset{-}{3}m$-PLGA, with a different amplitude. Moreover, a less intense band with an absorption maximum centered at 500 nm was obtained and, together with the previously reported shifts, suggested a global similar trend detected in ZB-PLGA, remarking a weaker interaction between the RS ZnO polymorph and PLGA.

### 3.4. Mechanical Properties of PLGA-ZnO Systems

**Figure 6:**
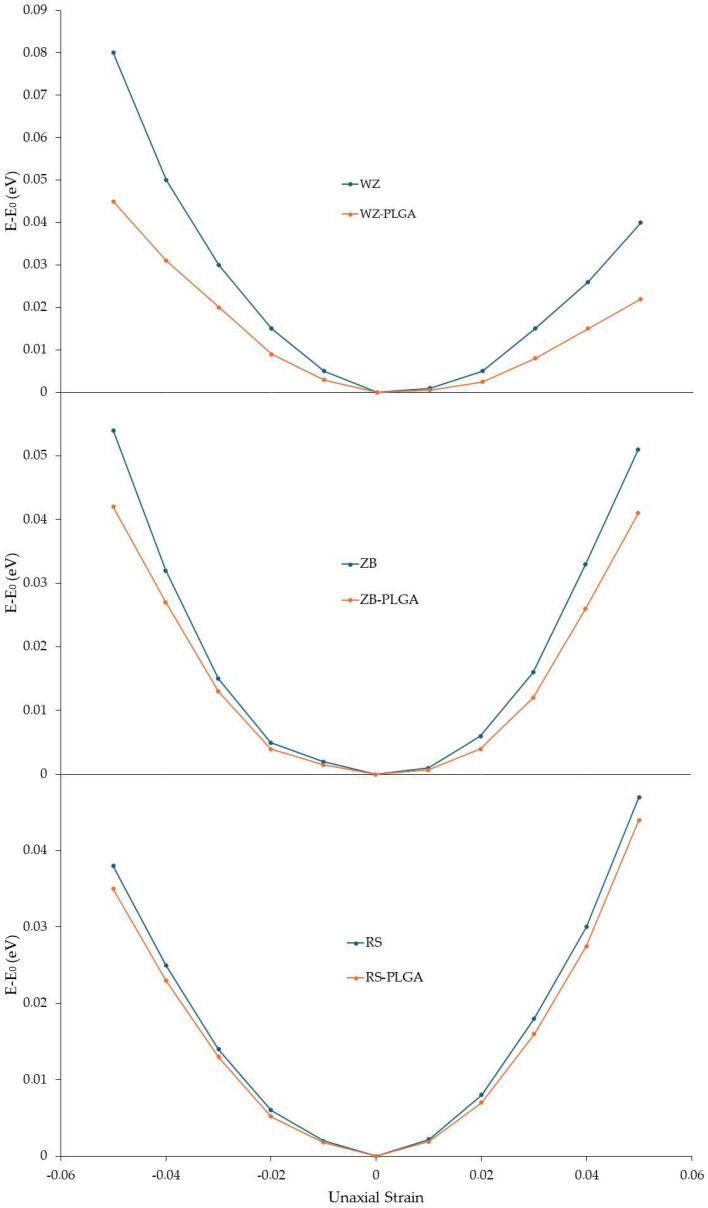
Strain energy under uniaxial loading for the function of the uniaxial strain of pure ZnO and ZnO with the adsorbed PLGA. The energy of the unstrained systems is reported to be zero.

After investigating the structural, electrical, and optical variations modulated by PLGA adsorption, the mechanical properties of the modeled ZnO-PLGA systems were calculated. In detail, Young’s in-plane moduli were computed for each system with and without a copolymer. To use Equation (8) reported in Section 2, the value of the ∂^2^E_s_/∂ε^2^ had to be calculated. Hence, for this purpose, the compressive and tensile strains were defined as negative and positive percentages, respectively. At the first step, the relaxed unit cell of each ZnO polymorph and each ZnO-PLGA surface was subjected to uniaxial compressive and tensile loading within the range of −5% to 5% while the strain steps were considered at 1%, and the calculated energies were recorded. The polymer was under strain as well. Then, the obtained energies were plotted with respect to the applied strain (Figure 6). Using this curve, ∂^2^E_s/_∂ε^2^ was calculated and substituted into Equation (8) to finally obtain the in-plane Young’s modulus. These results were plotted along the two in-plane directions, i.e., the x and y axes of the simulation boxes, alongside their second derivatives. Young’s computed moduli of the ZnO structures and adsorbed PLGA were reported (Table 3).

According to these results, PLGA adsorption decreased Young’s moduli of the whole system in both the x and y directions. In detail, the largest reduction was observed for WZ-ZnO, while the lowest reduction occurred for the RS polymorph in both directions. Moreover, the difference between the two directions was small, and this meant that the ZnO and the adsorbed PLGA structures had isotopic behavior; then, the adsorption was not influenced by this factor. The reported results are also in line with the findings related to electron density and PDOS.

The bulk modulus of each structure was also calculated, and the values in GPa are reported (Table 4). Also, in this case, the PLGA adsorption on the surface caused a decrease in the bulk moduli, meaning that a similar trend to Young’s modulus was observed for the bulk one, with the greatest and the lowest decreases observed for WZ-PLGA and RS-PLGA, respectively.

The electrons’ distribution increased in adsorbed PLGA, and the electron accumulation decreased among Zn atoms and increased around the O atoms. This decreased the bonding strength of Zn-Zn as well as Young’s and bulk moduli. Moreover, the bonding strength was even weaker between O and Zn in surface for the higher charge transfer compared with the other ZnO surfaces; therefore, the WZ-PLGA interface exhibited lower Young’s and bulk moduli. The semi-adsorbed PLGA structure on the ZB polymorph had less planar symmetry than WZ-PLGA. Therefore, fewer electrons participated in the xy plane, which resulted in weaker bonds between the components compared with the fully adsorbed PLGA on the WZ polymorph. This led to less charge transfer, which preserved a higher bonding strength between Zn-Zn and Zn-O. Consequently, a lower reduction in Young’s and bulk moduli in the ZB-PLGA systems was observed, and this was strictly correlated to the semi-adsorbed structure. In the case of the RS polymorph, even worse conditions for the PLGA adsorption were found.

The proposed method allowed simulating the behavior of the systems in their ground and excited states with high accuracy and in a reasonably short time, allowing us to understand which one had the best setup to be experimentally tested. The in silico approach that was proposed allowed us to reduce the time and costs of the laboratory and, therefore, to significantly decrease the amount of waste produced. The proposed methodology also drew guidelines for the experimental phase, which was, therefore, subsequent and more targeted. Furthermore, this method led to the investigation of systems in a pure or defective state, including doping agents or vacancy atoms, and the evaluation of how these defects can change the electrical, optical, and mechanical properties of the entire system.

On the other hand, since it is a first-principle full-atom approach, an important limitation of the method concerns the accurate treatment of every single atom present in the system, which becomes important when dealing with three-dimensional systems with more than 1000 atoms explicitly present. To overcome this problem, periodic boundary conditions were adopted, as discussed in Section 2, and the treatment of heavy systems requires important efforts in terms of computational costs, limiting its use. Since, in our case, we dealt with polymorphs, there were no problems of computational burdensomeness as each cell unit was repeated in the space with the boundary periodicity conductions, while as regards the PLGA, only short chains were included, still exploiting the periodic boundary conditions.

## 4. Conclusions

In this study, the adsorption of PLGA on different ZnO surfaces was deeply investigated to identify the capabilities of each ZnO polymorph to modulate the electrical, optical, and mechanical properties of the systems. Structural and electrical properties of the ZnO systems were calculated; then, the adsorption of PLGA was deeply analyzed for each inorganic system. From the three-dimensional displacements of the copolymer, the *P6₃mc* WZ ZnO surface showed a higher ability to absorb PLGA than the other two polymorphs, as confirmed by the calculation of the binding affinity and the bond lengths and angles at the interfaces. PDOS calculations for ZnO-PLGA systems identified an important increase in Coulomb potential with the WZ surface. Moreover, the charge transfer detected for this complex led to a variation in the PDOS spectra of the WZ polymorph before and after the PLGA interaction; then, the energy level also shifted due to the chemical interaction with the copolymer.

The absorption shifts induced by the PLGA interaction were identified to clarify the optical properties of ZnO polymorphs before and after the PLGA interaction. Indeed, while the absorption spectra of the three systems without PLGA seem in line with data from the literature and with the calculated bandgap, the adsorption degree of the copolymer led to evident variations. Signals attributed to WZ ZnO were no longer observed when the PLGA was adsorbed, while in other cases, they remained, and a gradual shift occurred. Finally, the mechanical properties in terms of Young’s and bulk moduli were simulated. The results obtained confirmed the highest decreases in both moduli after the PLGA adsorption on WZ ZnO, and the lowest effects were observed with the RS polymorph, in which the adsorption was not uniform or stable. To summarize, the best conditions to absorb PLGA in terms of stability, electrical, optical, and mechanical evidence were found with the use of WZ ZnO, promoting the use of this system for drug delivery and tissue engineering.

## Figures and Tables

**Table 1:** Lattice parameters of ZnO unit cells.

Polymorph	Space Group	Calculations	Experiments
Wurtzite (WZ)	*P6₃mc*	a = b = 3.250 Å c= 5.2060 Å	a = 3.258 Å c = 5.220 Å [33]
Zinc-Blende (ZB)	$F\overset{-}{4}3m$	a = b = 3.2402 Å c = 5.2204 Å	a = b = 3.1226 Å c = 5.1822 Å [34]
Rocksalt (RS)	$Fm\overset{-}{3}m$	a = b = c = 4.3420 Å	a = b = c = 4.294 Å [35]

**Table 2:** Binding affinity and bond lengths on surfaces.

Interface	Binding Affinity (eV)	Bond Lengths (Å)
WZ ZnO-PLGA	−1.92	1.42
ZB ZnO-PLGA	−0.61	1.37
RS ZnO-PLGA	0.03	/

**Table 3:** Young modulus values for the studied systems.

Structure	x	y
WZ	79.3 GPa [47]	78.9 GPa
WZ-PLGA	54.3 GPa	54.0 GPa
ZB	77.5 GPa	78.4 GPa
ZB-PLGA	64.6 GPa	65.5 GPa
RS	76.3 GPa	77.1 GPa
RS-PLGA	70.1 GPa	70.9 GPa

**Table 4:** Bulk modulus values for the studied systems.

Structure	Bulk Modulus	Difference (%)
WZ	222 GPa [48]	/
WZ-PLGA	142 GPa	36.04
ZB	151 GPa	/
ZB-PLGA	119 GPa	21.2
RS	116 GPa	/
RS-PLGA	108 GPa	6.9

## Data Availability

Data are contained within the article.
